# External Validation of the AS5F Score and the Role of Left Atrial Dilatation in Post-Stroke/TIA Atrial Fibrillation Detection

**DOI:** 10.3390/biomedicines14061378

**Published:** 2026-06-18

**Authors:** Aldo F. Costa, Rafael García, María J. Álvarez, Roberto Valverde, Isabel Pérez, Jerónimo Cruces, Pablo Doblas, Francisco J. Serrano, María L. Bustos, David Moreno, Claudia Ruz, Luis E. González, Víctor Lara, Cristóbal Muñoz, Eduardo Agüera-Morales

**Affiliations:** Department of Neurology, Reina Sofia Hospital, 14004 Córdoba, Spain; rafagr_lin@hotmail.com (R.G.); mjosefa.alvarez.sspa@juntadeandalucia.es (M.J.Á.); rvalverdemoyano@hotmail.com (R.V.); jeroparraga@gmail.com (J.C.); pablo.doblas98@gmail.com (P.D.); franciscordoba26@gmail.com (F.J.S.); bustosmorenomarialuisa@gmail.com (M.L.B.); davidmr2599@gmail.com (D.M.); claruzagu11@gmail.com (C.R.); luisgonzalezrnr@gmail.com (L.E.G.); 15mc07victorlara@gmail.com (V.L.); cristobalmm.2020@gmail.com (C.M.); fm2agmoe@uco.es (E.A.-M.)

**Keywords:** ischemic stroke, atrial fibrillation, left atrial dilatation, cardiac monitoring

## Abstract

**Background:** Prolonged cardiac monitoring increases atrial fibrillation (AF) detection after ischemic stroke or transient ischemic attack (TIA). The AS5F score was developed to identify patients at higher risk of post-stroke AF, but its performance in real-world populations remains incompletely characterized. We aimed to externally validate the AS5F score and to evaluate whether left atrial dilatation (LAD) improves risk prediction. **Methods:** We conducted a retrospective single-center study including 410 patients with ischemic stroke or TIA who underwent prolonged Holter monitoring between 2021 and 2025. Logistic regression and receiver operating characteristic (ROC) curve analyses were used to assess discrimination. Model calibration was evaluated using the Hosmer–Lemeshow test. **Results:** AF was detected in 33 patients (8.0%). The AS5F score was significantly associated with AF detection (OR 1.07 per point; 95% CI 1.03–1.11; *p* < 0.001), showing modest discrimination (AUC 0.69). Age alone demonstrated similar performance (AUC 0.69). LAD was strongly associated with AF (OR 4.00; 95% CI 1.79–8.93; *p* = 0.001) but had lower discriminatory ability (AUC 0.61). In patients with available echocardiographic data (*n* = 369), a combined age + LAD model achieved an AUC of 0.73 with adequate calibration. The improvement compared with AS5F was not statistically significant. **Conclusions:** In this external real-world cohort, AS5F demonstrated moderate discrimination for post-stroke AF detection. A simplified model combining age and left atrial dilatation showed numerically higher performance and may represent a pragmatic strategy for risk stratification in clinical practice.

## 1. Introduction

Atrial fibrillation (AF) is a leading cause of ischemic stroke and is frequently undiagnosed at the time of the index cerebrovascular event. Prolonged cardiac rhythm monitoring significantly increases AF detection, as demonstrated in randomized trials such as CRYSTAL AF and EMBRACE [1,2]. However, systematic long-term monitoring of all patients is not always feasible in routine clinical practice, highlighting the need for reliable risk-stratification strategies.

Among the proposed predictive tools, the AS5F score was developed using clinical and imaging variables independently associated with post-stroke AF detection. Although it demonstrated good discrimination in the derivation cohort, external validation in independent real-world populations remains limited, and predictive performance may decline when applied outside the original development setting.

Beyond clinical factors, structural atrial remodeling has emerged as a central pathophysiological substrate for AF. Left atrial dilatation (LAD) reflects chronic pressure and volume overload, atrial fibrosis, and electrical instability, all of which promote arrhythmogenesis [3,4]. The incorporation of simple structural markers obtained by transthoracic echocardiography may improve the identification of patients most likely to benefit from prolonged monitoring.

This study aimed to externally validate the AS5F score in a retrospective cohort of patients with ischemic stroke or transient ischemic attack (TIA) undergoing prolonged Holter monitoring and to assess the incremental predictive value of LAD combined with age in a parsimonious model.

## 2. Materials and Methods

### 2.1. Study Design and Population

We conducted a retrospective observational study of patients admitted with acute ischemic stroke or TIA who underwent prolonged Holter monitoring for AF detection between January 2021 and December 2025. Patients included in the present study had previously undergone prolonged Holter monitoring as part of routine clinical practice in cases of suspected cryptogenic stroke or TIA after standard etiological evaluation. The indication for prolonged monitoring had been established by the treating stroke physicians and was independent of the present retrospective analysis. Standard etiological evaluation included vascular imaging with computed tomography (CT) or magnetic resonance (MR) angiography, together with cardiac rhythm assessment using stroke unit telemetry for at least 24 h and/or a conventional 24 h electrocardiogram (ECG) Holter monitor. Consequently, patients with an already identified major atherosclerotic mechanism or previously documented AF were generally not considered candidates for prolonged Holter monitoring.

The AS5F score was calculated retrospectively for research purposes and was not used to guide monitoring indication during the study period. Patients with incomplete relevant clinical or imaging data were excluded. In addition, patients with less than 15 days of monitoring were also excluded in accordance with international recommendations for this monitoring modality. The study followed TRIPOD recommendations for reporting prediction model validation studies.

### 2.2. Outcome Definition

The primary outcome was the detection of AF during prolonged Holter monitoring. AF was defined by standard criteria: irregular R–R intervals (in the absence of atrioventricular conduction abnormalities), absence of discernible P waves, and irregular atrial activation lasting at least 30 s [5]. A wearable vest-based Holter monitoring device (Nuubo^®^, Valencia, Spain) was used, providing up to 30 days of continuous ECG monitoring. ECG recordings were analyzed by an experienced cardiologist, who reviewed and interpreted all studies.

### 2.3. Clinical and Echocardiographic Variables

Demographic data, clinical characteristics, neuroimaging findings, and echocardiographic parameters were extracted from the medical records. For echocardiographic parameters, all available data from each report was collected.

In most patients, echocardiographic information was reported qualitatively (e.g., presence or absence of LAD), whereas in others, more detailed quantitative measurements were provided. Additional echocardiographic parameters potentially associated with atrial remodeling and AF risk, such as left ventricular systolic or diastolic dysfunction, were not consistently available and therefore could not be incorporated into the present analysis.

The AS5F score was originally derived from variables associated with post-stroke or post-TIA AF detection. In the present study, the score was calculated according to the original publication as follows: age × 0.76 points, plus 9 points for a National Institutes of Health Stroke Scale (NIHSS) score ≤ 5 or 21 points for NIHSS > 5 [6].

### 2.4. Statistical Analysis

Continuous variables are presented as mean ± standard deviation or median (interquartile range [IQR]) and were compared using Student’s *t*-test or Mann–Whitney test as appropriate. Categorical variables are expressed as counts and percentages and compared between groups using X^2^ or Fisher’s test as appropriate.

Given the limited number of AF events, multivariable modeling was restricted to two predictors to avoid overfitting. Logistic regression analysis was performed to assess predictors of AF detection. Separate models were constructed for (1) the AS5F score, (2) age alone, (3) LAD alone, and (4) a combined model including age and LAD (age + LAD). Odds ratios (OR) with 95% confidence intervals (CI) were calculated. Given the limited number of AF events, multivariable models were intentionally restricted to a small number of predictors to minimize overfitting and preserve model stability.

Discriminative performance was evaluated using the area under the receiver operating characteristic curve (AUC). Comparisons between AUCs were performed using DeLong’s test. Model calibration was assessed using the Hosmer–Lemeshow goodness-of-fit test (5 groups). A two-sided *p*-value < 0.05 was considered statistically significant. All analyses were performed using Stata, version 16.0 (StataCorp LLC, College Station, TX, USA).

## 3. Results

Between January 2021 and December 2025, 451 patients underwent prolonged Holter monitoring. After excluding 23 patients with incomplete data, 3 who declined device placement, and 15 with less than 15 days of monitoring, 410 patients with ischemic stroke or TIA were included in the final analysis. AF was detected in 33 patients (8.0%).

The median age was 67 years (IQR 59–76), and 58.4% were male. The median time from the index event to device placement was 126 days (IQR 53–244), and the median monitoring duration was 26 days (IQR 22–27). It is notable that among patients in whom AF was detected, 55.2% had their first AF episode on the first day of monitoring. The median AS5F score was 62 (IQR: 55–68) and was significantly higher in patients with AF than in their counterparts (68 points [IQR: 63–73] vs. 61 points [IQR: 55–68], *p* < 0.001). Baseline characteristics are summarized in Table 1.

The AS5F score was significantly associated with AF detection (OR 1.07 per point; 95% CI 1.03–1.11; *p* < 0.001), demonstrating modest discriminative performance (AUC 0.69). Age was significantly associated with AF detection (OR 1.07 per year; 95% CI 1.03–1.12; *p* < 0.001), yielding an AUC of 0.69.

Echocardiographic data on left atrial size were available in 369 patients (90%). LAD was present in 40 patients (10.8%) and was strongly associated with AF detection in univariable analysis (OR 4.00; 95% CI 1.79–8.93; *p* = 0.001), although its discriminative ability was lower (AUC 0.61).

**Figure 1:**
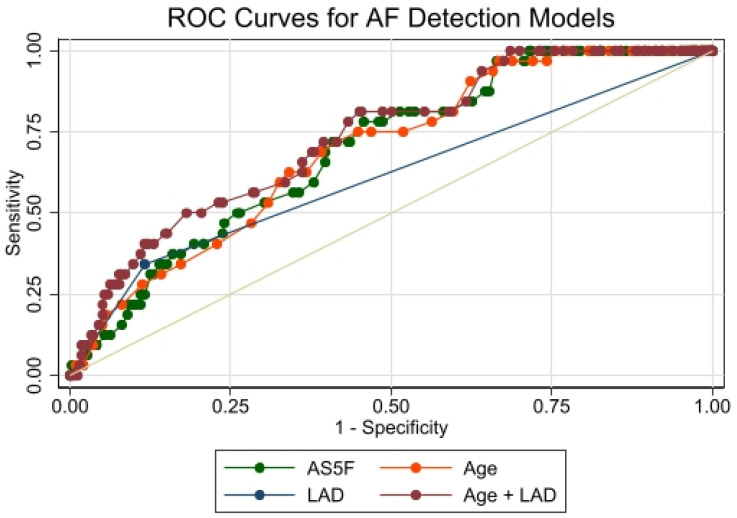
Receiver operating characteristic (ROC) curves for atrial fibrillation detection. Comparison of the AS5F score, age, and left atrial dilatation (LAD) alone, and a combined model including age and LAD in patients with complete echocardiographic data. The diagonal reference line (yellow line) represents the line of no discrimination, indicating performance equivalent to chance.

In the 369 patients with complete echocardiographic data, a multivariable logistic regression model including age and left atrial dilatation showed that both variables remained independent predictors of AF detection: age (OR 1.07; 95% CI 1.02–1.11; *p* = 0.002) and LAD (OR 2.93; 95% CI 1.27–6.75; *p* = 0.011). This combined model achieved an AUC of 0.73 (95% CI 0.65–0.82). When compared with the AS5F score (AUC 0.69), the combined model showed a numerical improvement in discrimination (ΔAUC ≈ 0.04), although this difference did not reach statistical significance (*p* = 0.24) (Figure 1). Model calibration assessed by the Hosmer–Lemeshow test (five groups) showed no evidence of poor fit (χ^2^ = 3.70, *p* = 0.296). Given the limited number of AF events, additional graphical calibration analyses were considered potentially unstable; therefore, model calibration was assessed using the Hosmer–Lemeshow goodness-of-fit test.

## 4. Discussion

The overall AF detection rate in our cohort was 8.0%, lower than rates reported in several prospective studies using early and protocolized prolonged monitoring strategies after cryptogenic stroke. For example, the CryptoAF registry, which used the same Nuubo^®^ textile wearable Holter device initiated within the acute phase of stroke, reported AF detection rates exceeding 20% after 28 days of monitoring [7]. Several factors may explain these differences. First, our cohort reflects a real-world clinical population without a fully standardized selection protocol for prolonged monitoring. Second, monitoring initiation occurred substantially later after the index cerebrovascular event (median 126 days), whereas landmark studies such as CRYSTAL AF, EMBRACE, and CryptoAF implemented rhythm monitoring much earlier after stroke onset. Delayed monitoring initiation may reduce AF detection yield, particularly for transient early post-stroke arrhythmias, and may partially contribute both to the lower AF prevalence and to the modest discriminative performance observed in our cohort.

Previous studies have proposed AS5F cutoff values around 67.5 for AF risk stratification; however, threshold performance may vary according to population characteristics and monitoring strategies. In the present study, we focused primarily on overall discriminative performance rather than on deriving an optimal cutoff.

In our study, the AS5F score demonstrated moderate discriminative performance for AF detection after ischemic stroke or TIA (AUC 0.69). Importantly, age alone showed nearly identical discrimination, suggesting that a substantial proportion of the score’s predictive ability may be primarily driven by age. The near-equivalent discrimination observed between age alone and AS5F raises the possibility that age captures a substantial proportion of the predictive information embedded within the score in real-world cohorts. A possible explanation may relate to the relatively low stroke severity observed in our cohort. The AS5F score incorporates NIHSS because cardioembolic strokes associated with AF are traditionally linked to larger infarcts and more severe neurological deficits. However, in our cohort, overall stroke severity remained relatively mild. In this setting, the discriminative contribution of NIHSS may have been attenuated, potentially reducing the incremental predictive value of AS5F beyond age alone.

LAD emerged as an independent predictor of AF detection and was associated with an approximately threefold increase in odds in multivariable analysis. Although LAD alone showed limited discrimination, its integration with age improved overall model performance (AUC 0.73) with adequate calibration, although without statistically significant improvement over AS5F.

Our findings are consistent with previous external validations. In the NOR-FIB cohort, AS5F showed acceptable discrimination (AUC approximately 0.72–0.74) [8]. Lee et al. reported an AUC of 0.689 [9], remarkably similar to our results. Hsieh et al. described a C-index of 0.73 in a Cox regression framework [10]. Although the C-index and AUC are not identical metrics, both assess discriminative ability, and differences across studies may reflect variations in study design, monitoring duration, and population characteristics. Overall, available evidence suggests that AS5F maintains moderate but consistent performance in routine clinical settings.

From a pathophysiological perspective, these findings are biologically coherent. Structural atrial remodeling reflects fibrosis, hemodynamic stress, and electrical instability that facilitate AF initiation and maintenance. The concept of atrial cardiopathy further expands this framework, proposing that atrial disease may predispose to embolic stroke even before overt AF is documented [11]. In this context, echocardiographic markers such as LAD may capture part of this underlying arrhythmogenic substrate and complement purely clinical predictors.

From a practical standpoint, decisions regarding prolonged rhythm monitoring after stroke are often individualized and resource-dependent. A simplified model combining age and an easily obtainable structural parameter may represent a pragmatic alternative to more complex risk scores, particularly when rapid bedside risk assessment is required.

This study has limitations, including its retrospective, single-center design, the moderate number of AF events, and incomplete echocardiographic data in a subset of patients that may limit generalizability to broader stroke populations. Additionally, the relatively long interval between the index event and monitoring initiation may have influenced AF detection rates.

## 5. Conclusions

In this real-world external cohort, the AS5F score demonstrated moderate discrimination for post-stroke AF detection, with a substantial contribution from age. The addition of a simple structural marker, such as left atrial dilatation, showed numerically higher discrimination, although without statistically significant improvement over AS5F. This suggests that integrating clinical and structural variables may enhance risk stratification in patients considered for prolonged rhythm monitoring. These findings should be interpreted cautiously, given the retrospective single-center design, moderate sample size, and relatively limited number of AF events, all of which may reduce statistical robustness and limit generalizability to broader stroke populations.

## Figures and Tables

**Table 1:** Demographic and clinical characteristics of the study population and prolonged Holter monitoring parameters.

Characteristics	Total Sample (*N* = 410)	Atrial Fibrillation (*n* = 33)	No Atrial Fibrillation (*n* = 377)	*p*-Value
**Age, median (IQR)**	67 (59–76)	75 (69–80)	67 (58–76)	<0.001
**Male sex, n (%)**	240 (58.4)	19 (57.6)	221 (58.6)	0.907
**NIHSS at admission, median (IQR)**	2 (0–5)	4 (1–7)	2 (0–5)	0.151
**Hypertension, n (%)**	256 (62.4)	20 (60.6)	236 (62.6)	0.821
**Diabetes mellitus, n (%)**	119 (29.0)	8 (24.2)	111 (29.4)	0.528
**Dyslipidemia, n (%)**	186 (45.8)	18 (54.6)	168 (45.0)	0.294
**Coronary artery disease, n (%)**	35 (8.5)	2 (6.1)	33 (8.8)	0.596
**Current or former smoking, n (%)**	107 (26.1)	7 (2.1)	100 (26.5)	0.505
**Chronic kidney disease, n (%)**	30 (7.3)	4 (12.1)	26 (6.9)	0.286
**Days to device implantation, median (IQR)**	126 (53–244)	122 (60–211)	128 (53–247)	0.616
**Monitoring duration (days), median (IQR)**	26 (22–27)	26 (22–28)	26 (22–27)	0.818
**Days to first AF episode, median (IQR)**	–	1 (1–6)	–	–
**Number of AF episodes, median (IQR)**	–	61 (12–506)	–	–
**Minutes in AF, median (IQR)**	–	76 (28–2645)	–	–
**AF burden (%), median (IQR)**	–	0.8 (0.1–10.2)	–	–
**AS5F score, median (IQR)**	62 (55–68)	68 (63–73)	61 (55–68)	<0.001
LAD, (proportions) *	50/369	11/32	39/337	<0.001

Abbreviations: National Institute of Health Stroke Scale, NIHSS; AF, atrial fibrillation; LAD, left atrial dilatation. * Echocardiogram information was available in 369 of 410 patients. Proportions are calculated based on those 369 patients, and the *p*-value corresponds to the χ^2^ test.

## Data Availability

All data reported in this paper will also be shared by the lead contact upon request.

## Abbreviations

The following abbreviations are used in this manuscript:

AF	Atrial fibrillation
TIA	Transient ischemic attack
LAD	Left atrial dilatation
ROC	Receiver operating characteristic
AUC	Area under the curve
CRYSTAL AF	The cryptogenic stroke and underlying AF
EMBRACE	30-day cardiac event monitor belt for recording atrial fibrillation after a cerebral ischemic event
NOR-FIB	The Nordic atrial fibrillation and stroke study
NIHSS	National Institute of Health Stroke Scale
IQR	Interquartile range
OR	Odds ratio
CI	Confidence interval
